# Comparative analysis of pulmonary function decline in patients undergoing bronchoscopic lung volume reduction with endobronchial valves versus conservative treatment in emphysema management: A longitudinal coarsened exact matched analysis

**DOI:** 10.1177/14799731251350709

**Published:** 2025-06-11

**Authors:** Johannes Wienker, Kaid Darwiche, Rüdiger Karpf-Wissel, Dirk Westhölter, Erik Büscher, Sebastian Zensen, Johannes Haubold, David Kersting, Hubertus Hautzel, Josef Homola, Christian Taube, Marcel Opitz, Marc Struß

**Affiliations:** 1 Department of Pulmonary Medicine, Division of Interventional Pneumology, 530897 University Medicine Essen-Ruhrlandklinik, Essen, Germany; 2 Department of Pulmonary Medicine, 530897 University Medicine Essen-Ruhrlandklinik, Essen, Germany; 3 Institute of Diagnostic and Interventional Radiology and Neuroradiology, 39081 University Hospital Essen, Essen, Germany; 4 Department of Nuclear Medicine, 39081 University Hospital Essen, Essen, Germany

**Keywords:** chronic obstructive pulmonary disease, emphysema, bronchoscopic lung volume reduction, valves, longitudinal data

## Abstract

**Background:**

Chronic obstructive pulmonary disease (COPD) and emphysema display a chronic and progressive disease for the individual patient. The forced expiratory volume in one second (FEV_1_) is declining with age as displayed in the Fletcher–Peto curve. Despite established benefits of bronchoscopic lung volume reduction (BLVR) using endobronchial valves (EBVs), long-term data suggest a gradual reduction in the magnitude of these benefits.

**Purpose:**

This study aimed to compare the rate of lung function change in emphysema patients undergoing BLVR versus those receiving conservative management, utilizing coarsened exact matching to ensure balanced baseline characteristics.

**Patients and Methods:**

In this retrospective single center study data between 2015 and 2021 was analyzed. BLVR patients achieving significant volume reduction (≥563 mL) were matched to conservatively managed controls based on age, sex, BMI, and smoking history. Pulmonary function changes after successful BLVR with valves, including forced expiratory volume in one second (FEV_1_) and residual volume (RV), were monitored and analyzed over a 3-year period.

**Results:**

A total of 60 patients, evenly distributed between the two groups (30 each), were included in the analysis. Median FEV_1_ change was −0.063 L/year for BLVR patients and −0.066 L/year for controls. No statistically significant differences in annual FEV_1_ and RV changes were observed (−0.07 vs −0.08, *p* = 0.492; −0.07 vs −0.07, *p* = 0.569; −0.05 vs −0.04, *p* = 0.636 at follow-ups in years 1, 2, and 3, respectively for FEV_1_ and +0.20 vs +0.25, *p* = 0.643; +0.80 vs +0.65, *p* = 0.960; +1.0 vs +0.85, *p* = 0.963 at follow-ups in years 1, 2, and 3, respectively for RV).

**Conclusion:**

In this matched cohort analysis, no significant differences in annual changes in FEV_1_ or RV progression were observed between patients after successful BLVR with valves and patients under conservative treatment. The results indicate that COPD progression is the main factor for the decline in functional improvement after successful BLVR with valves.

## Introduction

Chronic obstructive pulmonary disease (COPD) and emphysema are mainly smoking-related disorders and affect millions of people worldwide, with a large effect on individual patients and society as a whole.

The generally accepted idea that forced expiratory volume in 1-s (FEV_1_) decline accelerates with age is reflected in the Fletcher–Peto curve, a model proposed by Fletcher and Peto illustrating lung function decline across an individual’s lifespan.^1^ More recent evaluations show that lung function decline is a feature in COPD patients, however different trajectories of decline have been defined.^2^

FEV_1_ decline in COPD patients generally progresses more rapidly than in smokers without COPD, although recent studies highlighted considerable variability in the rate of decline among COPD patients. In the BODE Cohort, emphysema patients exhibited a significant FEV_1_ decline ranging from −32 to −278 mL/yr, with higher baseline FEV_1_ and low body mass index emerging as independent factors associated with accelerated decline.^3^

Bronchoscopic lung volume reduction (BLVR) has been thoroughly investigated through multiple randomized clinical trials and is now an established standard treatment for patients with severe emphysema in specialized centers.^4,5,6^

While long-term data have confirmed sustained improvements in lung function and quality of life following endobronchial valve (EBV) treatment, the magnitude of these benefits diminishes gradually over time.^7,8^ However, it has not been thoroughly investigated if BLVR results in an accelerated decline in lung function compared to the natural decline.

In this study, we aim to compare the lung function decline of emphysema patients who underwent BLVR indicating with those who received conservative treatment, utilizing a coarsened exact matching approach. To address this question meaningfully, we included only patients in the BLVR group who achieved significant target lobe volume reduction (TLVR), as this response reflects a successful interventional procedure. Restricting the analysis to responders enables a valid comparison with conservatively treated patients and minimizes confounding from ineffective or incomplete interventions.

## Methods

### Patients

We conducted a retrospective study involving patients with severe emphysema who were evaluated for treatment with EBVs for lung volume reduction at Ruhrlandklinik, University Medicine Essen, Germany, from 2015 to 2021.

We then identified all patients who did not receive BLVR and were clinically monitored and received regular pulmonary function measurements in our clinic over a course of at least 3 years. The first follow-up after BLVR was conducted 3 months post-intervention and was defined as the baseline for subsequent comparisons. Additional follow-up assessments were performed at 12, 24 and 36 months after the intervention for the BLVR group or after the initial clinic visit for patients receiving conservative treatment. In addition to smoking cessation, conservative treatment comprised optimal pharmacological therapy, regular exercise and physiotherapy. Pulmonary function assessments were performed using body plethysmography and spirometry according to ERS/ATS guidelines.

We employed coarsened exact matching (CEM) to create comparable groups of patients undergoing BLVR treatment. This displays a statistical technique designed to reduce imbalance in observational studies by matching treatment and control groups on coarsened versions of covariates. In CEM, variables are temporarily grouped into meaningful categories (“coarsened”) before matching, allowing for greater flexibility and improved covariate balance compared to exact matching. This method is particularly advantageous in smaller datasets, as it retains more observations while still controlling for confounding.^9^

The matching criteria included age, sex, body mass index (BMI), pack years of smoking to ensure balanced baseline characteristics between patients and controls. For age and BMI, we arbitrarily selected 50, 60, and 70 years as age cut-points, and 18.5, 25, and 30 as BMI thresholds to classify underweight, normal weight, and overweight, respectively. As BMI is a composite variable that reflects both height and weight, we used BMI alone as a matching criterion in our CEM procedure. Height and weight were not matched separately to avoid redundancy and preserve an adequate sample size. Among treated patients, only those who achieved clinically relevant volume reduction (MCID – minimally clinically important difference = 563 mL)^10^ were included in the final cohort which was then matched to the non-interventionally treated controls. MCID was assessed by measuring target lobe volume reduction (TLVR), based on pre- and post-treatment quantitative CT analyses, which provided detailed, lobar-specific volumetric data. This patient selection ensures a valid comparison between effective intervention and conservative treatment and eliminates confounding from suboptimal or failed BLVR procedures. Patients with major comorbidities, such as severe cardiovascular disease, active malignancy or orthopedic immobility, were excluded from the study.

The study was conducted in compliance with the guidelines of the Institutional Review Board of the University Hospital Essen. The Ethics Committee of the University of Duisburg-Essen (Approval Number 24-12294-BO) waived the informed consent due to the retrospective and anonymous nature of this study. The data were completely anonymized before being included in the study.

### Statistics

We created matched groups in a one-to-one approach based on covariates, including age, gender, body mass index and pack years. The suitability of CEM was evaluated through pre- and post-match imbalance assessments. Overall imbalance was measured using the L1 statistic, as described by Iacus et al.^11^ To assess the quality of balance before and after matching, the L1 multivariate imbalance measure was calculated. The L1 decreased from 0.28 before matching to 0.0 after matching, denoting a perfect match. We assessed differences between the two cohorts for each variable using chi-square tests and comparisons of empirical quantiles. There were no missing values for the primary outcome measures.

Data preprocessing for matched group generation was performed using R (R Foundation for Statistical Computing, Vienna, Austria), SPSS (IBM Corp., Armonk, NY), and the CEM-Extension bundle (Matthew Blackwell). All statistical analyses were conducted in SPSS version 23. Variables were assumed to follow a non-normal distribution and are presented as median and range, with group comparisons carried out using Mann-Whitney U tests.

## Results

Between January 2015 and October 2021, patients were systematically screened for inclusion in this analysis. A total of 324 patients with a median age of 61.5 years (range: 50 – 76 years) underwent EBV-treatment at our center. Of these, 89 patients (27.5%) did not achieve the minimal clinically important difference (MCID) threshold of 563 mL for significant volume reduction and were therefore excluded from further analysis.

**Figure 1.**
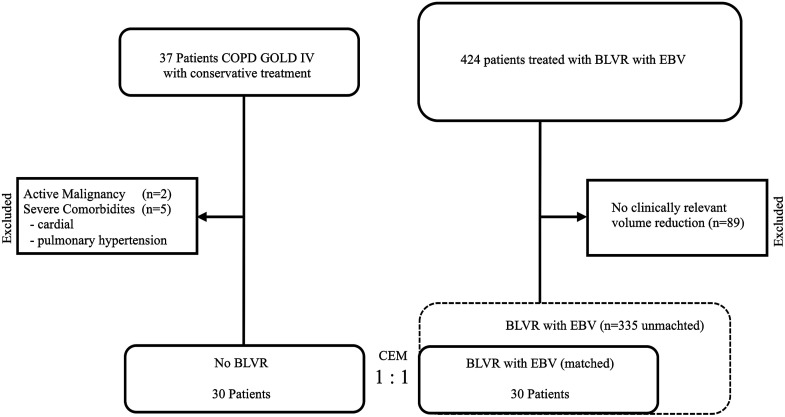
Data availability flow chart. BLVR, bronchoscopic lung volume reduction; EBV, endobronchial valves; CEM, coarsened exact matching.

In the non-interventional (conservative) treatment group, 37 patients were identified, with a median age of 62 years (range: 46 – 74 years). Seven patients were excluded due to severe comorbidities, including pulmonary hypertension, orthopedic immobility, cardiac insufficiency (n = 5) or active malignancy (n = 2). The reasons for continuing conservative therapy included personal reluctance toward BLVR, lung hyperinflation marginally below the clinic-specific cutoff value (RV < 200%) and lack of fissure integrity (Figure 1).

The baseline demographic and clinical data of the 30 matched patients in the intervention and 30 controls are displayed in Table 1. All patients showed severe airway obstruction and hyperinflation. In patient with BLVR the median FEV_1_ increased from 0.73 L to 0.87 L and RV decreased from 5.90 L to 4.50 L following the intervention. In the time after the intervention the control patients showed a decline in FEV_1_ of 0.08 L in the year 1, 0.15 L in year two and 0.19 L in year 3 with a rate of 0.066 L / year. Patients who underwent BVLR showed an decline in FEV_1_ of 0.07 L in the year 1, 0.14 L in year two and 0.19 L in year 3 with a rate of 0.063 L / year.

**Table 1.** Demographic and clinical characteristics at baseline.

Parameter	Median (range) or number (no.)		*p*-value
	**BLVR**	**No-BLVR**	
Female/male, n	17/13	17/13	
Age, years	61.5 (50-76)	62.0 (46-74)	0.778
Weight, kg	64.0 (52–103)	65.0 (51–90)	0.631
Height, cm	168 (156–187)	170 (155–186)	0.394
Body mass index, kg/m^2^	22.2 (16–33)	23.0 (17–30)	0.965
Smoking history, pack-years	32.5 (25–90)	35.0 (30–90)	0.146
FEV_1_–pre BLVR			
Liters	0.73 (0.45– 1.1)	–	
% Predicted	25 (15–38)	–	
FEV_1_–post BLVR/Baseline			
Liters	0.87 (0.51–1.18)	0.78 (0.54–1.28)	0.240
% Predicted	31 (18–47)	31 (18–47)	0.463
RV–pre BLVR			
Liters	5.90 (3.61–11.6)	–	
% Predicted	266 (201–378)	–	
RV–post BLVR/Baseline			
Liters	4.50 (3.01–7.90)	5.15 (3.60–7.70)	0.264
% Predicted	221 (168–300)	218 (182–379)	0.559

Values are Median (Range) or Number (No).

FEV_1_, forced expiratory volume in 1 s; RV, residual volume; BLVR, bronchoscopic lung volume reduction. Statistically significant differences between groups tested with Mann–Whitney-test.

RV increased over time in control and BLVR group with an annual increase of 0.28 L and 0.33 L respectively. The BLVR group received a standard 3-day peri-interventional antibiotic prophylaxis. Patients of both groups continued a guideline-based pharmacological therapy. There were no deaths, no severe complications postinterventionally and no instances of valve removal observed during the study period.

**Figure 2.**
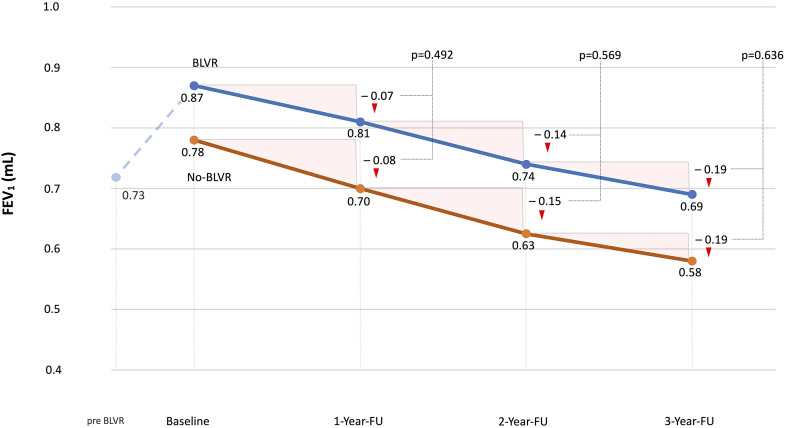
FEV_1_ over a 3-year follow up interval. Both the BLVR and No-BLVR patient groups exhibited a comparable trajectory of FEV_1_ decline, with no statistically significant differences observed between the groups. BLVR, bronchoscopic lung volume reduction; FEV_1_, forced expiratory volume in 1 s; FU, follow-up.

The annual analyses demonstrated no statistically significant differences in FEV_1_ decline between the groups (– 0.08 L vs – 0.07 L, *p* = 0.492; – 0.07 L vs – 0.07 L, *p* = 0.569; – 0.04 L vs – 0.05 L, *p* = 0.636 at follow-ups in years 1, 2, and 3, for No-BLVR vs BLVR respectively). Similarly, the progression of hyperinflation, as indicated by changes in residual volume (RV), showed no significant differences between the two groups (+ 0.25 L vs + 0.20 L, *p* = 0.64; + 0.65 L vs + 0.80 L, *p* = 0.96; + 0.85 L vs + 1.0 L, *p* = 0.96 at follow-ups in years 1, 2, and 3, respectively) as displayed in table 2 and Figure 2.

**Table 2.** Pulmonary function over a 3-year follow-up interval.

	Parameter	Baseline	Follow-up (1-year)	p-value	Follow-up (2-year)	p-value	Follow-up (3-year)	p-value
BLVR	FEV_1_, L	0.87 (0.51–1.18)	0.81 (0.45–1.10)		0.74 (0.38–1.04)		0.69 (0.36–0.99)	
	ΔFEV_1_, L	–	−0.07^*^	0.492^*^	−0.14^**^	0.569^**^	−0.19^***^	0.636^***^
	RV, L	4.5 (3.0–7.9)	4.7 (3.2–8.1)		5.3 (3.5–8.4)		5.5 (3.7–8.6)	
	ΔRV, L	–	+0.20^†^	0.643^†^	+0.80^††^	0.960^††^	+1.0^†††^	0.963^†††^
No-BLVR	FEV_1_, L	0.78 (0.54–1.28)	0.70 (0.46–1.30)		0.63 (0.39–1.21)		0.58 (0.38–1.26)	
	ΔFEV_1_, L	–	−0.08^*^	0.492^*^	−0.15^**^	0.569^**^	−0.19^***^	0.636^***^
	RV, L	5.2 (3.6–7.7)	5.4 (3.8–7.9)		5.8 (4.1–8.2)		6.0 (4.3–8.4)	
	ΔRV, L	–	+0.25^†^	0.643^†^	+0.65^††^	0.960^††^	+0.85^†††^	0.963^†††^

FEV_1_, forced expiratory volume in 1 s; RV, residual volume.

Mann-Whitney-Test for differences between groups marked.

## Discussion

This study analyzed patients with severe emphysema undergoing either BLVR with endobronchial valves or receiving non-invasive conservative management. Patients in the BLVR group were included only if they achieved a clinically meaningful reduction in lung volume, while patients were generally excluded if they had severe comorbidities. Median lung function changes over 3 years showed no statistically significant differences in the rates of decline in FEV_1_ or progression of hyperinflation (residual volume) between the two groups.

Our findings demonstrate that the benefits of BLVR with EBV in enhancing pulmonary function can persist for at least 2 years, with values remaining above baseline levels throughout this period. These results align with previously reported research outcomes.^12^ COPD is well established as a chronic, progressive condition that continues to advance even after smoking cessation. Tantucci et al. reviewed 10 studies examining the decline in pulmonary function across various stages of COPD, reporting annual FEV_1_ reductions ranging from 35 mL/year to 79 mL/year, with the most pronounced declines observed in the early stages of the disease.^13^ This finding contrasts with the Fletcher-Peto analysis, which suggests accelerated FEV_1_ decline later in life and in more advanced disease stages.^1^ However, Lange et al. highlighted that COPD can develop through multiple lung-function trajectories, including impaired lung growth during early life, resulting in low peak lung function, or an accelerated decline in lung function in adulthood despite achieving normal peak lung function. Patients therefore may already be predisposed due to suboptimal lung development or additional risk factors such as smoking.^2^ In the present investigation, all patients were classified as GOLD stage IV, presenting with severe hyperinflation due to extensive emphysematous destruction of lung parenchyma. The annual FEV_1_ decline in this cohort ranged from 50 mL/year to 80 mL/year, exceeding the rates reported in the earlier mentioned review. Consistent with these findings, the Evaluation of COPD Longitudinally to Identify Predictive Surrogate Endpoints (ECLIPSE) study by Vestbo et al. demonstrated significantly greater FEV_1_ decline in patients with CT-confirmed emphysema compared to those without emphysematous changes.^14^

The observed decline in lung function in both BLVR and non-BLVR groups reflects the natural course of severe emphysema, which remains progressive even after bronchoscopic intervention or optimized medical therapy. Endobronchial valve treatment primarily achieves a physiological improvement by inducing targeted lobar atelectasis, thereby reducing lung hyperinflation, restoring diaphragmatic function and improving mechanical efficiency of the respiratory muscles. However, the intervention does not address the underlying pathological loss of elastic recoil or the destruction of alveolar connective tissue. As such, while BLVR can temporarily improve lung mechanics and ventilation distribution, it does not modify the disease process itself and patients continue to experience gradual pulmonary function decline due to ongoing parenchymal degeneration. Disease progression has also been documented in other lung volume reduction treatment modalities, including lung volume reduction surgery (LVRS) and BLVR using coils. In these approaches, initial improvements in pulmonary function, as measured by FEV_1_, were observed. However, over time FEV_1_ declined returning to baseline levels approximately 2 years after the intervention. This is also consistent with our observational findings.^15,16^

Different factors have previously been identified to be responsible or even accelerate the disease progression in emphysema and COPD patients. These include next to continued exposure to noxious agents, a high frequency of exacerbations, chronic bronchitis or airway dysbiosis.^17,18,19^

Following BLVR with valves, compensatory hyperinflation of the untreated lobes has been reported to limit the duration of sustained improvements in lung function.^20^ Although not statistically significant, in the current study the BLVR group demonstrated a slightly greater progression in RV compared to the non-BLVR group, which may, to some extent, be attributed to the compensatory hyperinflation observed after BLVR.

Parallel to our observations, Hartmann et al. compared pulmonary function trends before and after BLVR, demonstrating that the treatment did not alter the rate of FEV_1_ decline.^21^ However, in contrast to this study, Hartmann et al. did not exclude patients not achieving the minimal clinically important difference threshold of a 563 mL lung volume reduction after BLVR.

There are several limitations to our study. First, the study is limited by a relatively small sample size, as the majority of patients receiving non-interventional treatment are typically monitored in an outpatient setting and therefore less frequently undergo standardized follow-up clinical assessment and lung function testing. Another is its retrospective design, conducted at a single emphysema care center. Additionally, data on quality of life and physical performance were not available for many patients. While we had complete data for all BLVR patients, such data were lacking for patients receiving conservative treatment, as they were primarily seen in an outpatient setting in which the 6-min walk test was not routinely performed. The absence of data on quality of life and physical performance limits the ability to fully assess the functional and patient-perceived benefits of BLVR, as improvements in lung function do not always correlate directly with enhanced daily functioning or well-being. Another limitation is that the analysis included only patients in the BLVR group who achieved the minimal clinically important difference (MCID) in volume reduction. Patients who underwent BLVR but did not meet this threshold were not included in the dataset. As a result, a broader evaluation of all treated patients was not possible, which may limit the generalizability of our findings. Future studies could benefit from incorporating this wider patient population in a sensitivity analysis.

In summary, our results demonstrate that BLVR improves lung function in patients with severe emphysema but does not affect the rate of disease progression. Furthermore, no accelerated decline in pulmonary function was observed following treatment. These findings highlight the progressive nature of the disease and provide evidence that BLVR does not contribute to an accelerated decline in pulmonary function.

## Data Availability

The datasets generated and analyzed during the current study are available from the corresponding authors on reasonable request.*
